# Dynamic DWI detects respiratory modulation of brain parenchymal hydrodynamics

**DOI:** 10.1162/IMAG.a.1369

**Published:** 2026-09-24

**Authors:** Jianing Zhang, Elodie Foster, Adam M. Wright, Xiaopeng Zhou, Qiuting Wen

**Affiliations:** Department of Radiology and Imaging Sciences, Indiana University School of Medicine, Indianapolis, IN, United States; Weldon School of Biomedical Engineering Department, Purdue University, West Lafayette, IN, United States; College of Health and Human Sciences, Purdue University, West Lafayette, IN, United States

**Keywords:** neurofluids, dynamic DWI, hydrodynamics, physiology, cardiac pulsation, coupling, respiration, parenchyma

## Abstract

The circulation of cerebrospinal fluid (CSF) within the brain parenchyma and its exchange with interstitial fluid (ISF) are essential for maintaining homeostasis and clearing interstitial waste. However, the physiological forces driving parenchymal hydrodynamics remain unclear. Cardiac pulsation alone may be insufficient to drive parenchymal flow, while respiration has emerged as an additional contributor. To date, however, most respiration-related studies have focused on the ventricular system and spinal canal, leaving its influence on parenchymal hydrodynamics—the site of waste production—largely unexplored. In this study, we investigated whether parenchymal hydrodynamics are coupled with respiration during normal breathing, and how this coupling compares with that driven by cardiac pulsation. We used dynamic diffusion-weighted imaging (dynDWI) with a b-value of 150 s/mm² to capture incoherent fluid motion, quantified using the apparent diffusion coefficient (ADC). Respiration and cardiac signals were simultaneously recorded using a respiratory belt and finger photoplethysmography (PPG). Temporal coupling strength and time delay between parenchymal hydrodynamics and physiological signals were assessed using TRACC-PHYSIO (Time-domain Resolution-Aligned Cross-Correlation) for physiological coupling. We found that temporal ADC fluctuations in the parenchyma were coupled to respiration, with a latency of 0.7 seconds, and exhibited a gray-to-white matter propagation pattern. Spatial coupling patterns differed between respiration and cardiac pulsation: respiration coupling was stronger in gray and white matter than in the perivascular subarachnoid space (PVSAS) and lateral ventricles (LV), whereas cardiac coupling predominated in the PVSAS and LV. Rebinning dynDWI into respiration cycles revealed parenchymal ADC peaked during inhalation and reached a minimum during exhalation. Together, these findings suggest that respiration contributes to driving parenchymal hydrodynamics and fluid-driving forces vary spatially across brain regions, with respiration and cardiac pulsation playing complementary roles in regulating brain fluid dynamics.

## 1 Introduction

Cerebrospinal fluid (CSF) transport is driven by several physiological pumps, including cardiac pulsation, respiration, and low-frequency oscillations (LFOs) (Rasmussen et al., 2022). However, the drivers of parenchymal hydrodynamics—particularly in the perivascular CSF and interstitial fluid (ISF) spaces—remain largely unclear in humans. The movement of perivascular CSF and its exchange with ISF hold great research interest because of its central role in flushing interstitial waste (Iliff et al., 2012). While cardiac pulsation drives CSF flow in the perivascular subarachnoid space (PVSAS) (Bedussi et al., 2018; Mestre et al., 2018), this force is substantially dampened by the high compliance and resistance of penetrating arterioles as it propagates into the parenchyma. Mechanical models also suggest cardiac alone may be insufficient to drive directional flow within the parenchyma (Kedarasetti et al., 2020; Piersanti et al., 2023; Sharp, 2023). In contrast, rodent studies have proposed alternative driving forces, such as LFOs, for CSF transport through parenchymal tissue (Hauglund et al., 2025; Holstein-Rønsbo et al., 2023; Jiang-Xie et al., 2024; Murdock et al., 2024; van Veluw et al., 2020).

Respiration has also emerged as an important driver of CSF transport, particularly in human studies. Most research to date has focused on its effects within the ventricular system and spinal canal, using flow-sensitive imaging techniques such as phase-contrast (PC) MRI (Chen et al., 2015; Dreha-Kulaczewski et al., 2015; Kollmeier et al., 2022; Lagana et al., 2022; Liu et al., 2025; Toger et al., 2022; Vinje et al., 2019), functional MRI (fMRI) (Nair et al., 2022, 2024), and spin-labeling methods (Wang et al., 2022; Yamada et al., 2013). These studies consistently demonstrate strong respiration-driven CSF oscillations during breathing tasks—such as paced breathing and breath holding—throughout the ventricles, prepontine cisterns, and cervical spinal canal. More recent investigations have extended to the subarachnoid space (SAS) using PC-MRI with slow-flow sensitivity (Dong et al., 2025), and to the perivascular space surrounding penetrating vessels using high-resolution 3D imaging with motion-sensitive gradients (Hirschler et al., 2025). These studies confirm that respiration contributes to CSF dynamics in both the SAS and perivascular space.

Within the parenchyma, however, it remains unclear whether respiration continues to drive CSF and ISF dynamics under normal breathing conditions. A key consideration in imaging parenchymal hydrodynamics is that fluid motion is no longer fast or coherent—as seen in the aqueduct or spinal canal—and thus cannot be reliably detected using conventional flow-sensitive techniques. In the small perivascular spaces, CSF moves at extremely slow velocities (typically less than 20 μm/s) (Bedussi et al., 2018; Mestre et al., 2018) and in nonuniform, multidirectional patterns. ISF transport further involves a combination of diffusion and convection, with their relative contribution depending on physiological conditions (Mestre et al., 2020; Rasmussen et al., 2022). Diffusion MRI is, therefore, well suited to capture such slow, randomly oriented fluid dynamics (Wright, Wu, Feng, et al., 2024). This sensitivity has been demonstrated in phantoms, where the apparent diffusion coefficient (ADC) reflects combined effects of diffusive and convective effects (Komlosh et al., 2019), and in animal studies showing increased parenchymal ADC following pharmacological enhancement of fluid transport with an AQP4 facilitator (Alghanimy et al., 2023). Collectively, these findings support diffusion imaging as a promising tool for probing parenchymal hydrodynamics and their physiological drivers.

In the present study, we use dynamic diffusion-weighted imaging (dynDWI) to capture the temporal dynamics of fluid movement within the brain parenchyma. Previous work by us and others has demonstrated that dynDWI, with carefully selected b-values, is sensitive to CSF mobility and its coupling with cardiac pulsation in the perivascular subarachnoid space (Han et al., 2024; Wen et al., 2022, 2023; Wright, Wu, Yang, et al., 2024) and within the parenchyma (Wen et al., 2025). Building on this foundation, the current study investigates whether parenchymal hydrodynamic signals also couple with respiration under normal breathing conditions, and how this coupling compares with that driven by cardiac pulsation.

## 2 Materials and Methods

### 2.1 Participant recruitment

We recruited 46 healthy participants (11 males, 35 females; mean age: 53.8 ± 12.8 years, range: 35 ~ 82 years) from the local community. All participants had no history of neurological, cardiovascular, or respiratory disorders. Written informed consent was obtained from each participant in accordance with the protocols approved by the institutional review boards (IRBs) at Indiana University.

### 2.2 Data acquisition

All scans were performed on a 3T Siemens Prisma scanner with a 64-channel head–neck coil. The dynDWI scan was acquired using a single-shot echo-planar imaging (EPI) sequence with Stejskal–Tanner pulsed gradient spin echo. Diffusion weighting was applied along three orthogonal directions (x/y/z) with a b-value of 150 s/mm^2^, ensuring sensitivity to incoherent CSF and ISF mobility while suppressing rapid blood flow in the arterioles and capillaries (Wen et al., 2022). Each diffusion direction was repeated 45 times to capture the fluid dynamics. Ten nondiffusion-weighted images (b-value = 0 s/mm^2^) were acquired and averaged for ADC calculation. The acquisition parameters were as follows: repetition time (TR)/echo time (TE) = 1750 ms/49.6 ms, multiband factor = 2, slice number = 36, matrix size = 134 × 134, voxel size = 1.8 × 1.8 × 4 mm^3^, total scan time = 4 minutes 58 seconds. During dynDWI scan, respiratory activity was recorded using the Siemens scanner’s built-in respiratory belt, and cardiac pulsation was monitored using the built-in finger plethysmography (PPG) sensor. Both physiological signals were recorded simultaneously with MR data within the physio-suite of the scanner. Temporal alignment was ensured through the scanner’s internal time-logging system, in which each slice-excitation pulse is associated with a corresponding timestamp in the physiological recording. This configuration enables high-precision retrospective synchronization between image acquisition and physiological traces.

T1-weighted anatomical images were acquired with a three-dimensional magnetization rapid gradient echo (MPRAGE) sequence. The acquisition parameters were TR/TE = 2300 ms/2.98 ms, FA = 9°, slice number = 256, matrix size = 240 × 240, voxel size = 1.0 × 1.0 × 1.0 mm^3^, total scan time = 5 minutes 30 seconds.

### 2.3 Data quality control

Quality control (QC) procedures were applied to both dynDWI and physiological recordings. Respiration and finger PPG were visually inspected, and recordings with excessive noise were excluded. Motion artifacts in dynDWI data were assessed using FSL’s MCFLIRT function. Absolute displacement of each DWI volume was calculated relative to a fixed reference volume, and relative displacement was computed between adjacent volumes. To avoid confounding effects from general motion and respiration-related motion, QC was conducted at two levels. First, participants with excessive general head motion—defined as absolute displacement >1 mm or relative displacement >0.35 mm in more than 15% of volumes—were excluded. Second, to further control respiration-related motion, we assessed the correlation between motion parameters and respiratory belt signals. Participants with a maximum correlation coefficient >0.3 were excluded (Supplementary Fig. S1). Based on these criteria, 14 participants were removed from analysis: 3 due to poor physiological data, 4 due to excessive head motion, and 7 due to respiration-related head motion. A total of 32 participants remained for the final analysis. The demographic information of the participants is summarized in Supplementary Table S1.

### 2.4 TRACC-PHYSIO

To quantify temporal coupling between dynDWI signals and respiration, we employed the Time-domain Resolution-Aligned Cross-Correlation method to estimate PHYSIOlogical coupling and time delays (TRACC-PHYSIO) (Wright, Zhang, et al., 2026). Unlike retrospective binning approaches that average signals into one cycle, TRACC-PHYSIO preserves the native time domain and enables quantitative estimation of both coupling strength and temporal delay by cross-correlating dynDWI signals (sampled at TR = 1750 ms) with high-resolution physiological recordings (400 Hz, 2.5 ms per sample). Leveraging the high sampling rate of the physiological recordings, TRACC-PHYSIO provides millisecond-level temporal resolution (2.5 ms) for estimating brain–peripheral delays, which in turn allows comparison of relative delays across brain regions. This framework has been shown to recover short cardiac impulse delays consistent with known cardiac propagation velocities and biomechanical modeling predictions, including brain-to-finger delay (Wen et al., 2023), arterial-to-venous delay (Wright, Xu, et al., 2025), and PVSAS-to-GM delay (Wen et al., 2025). Simulation studies further demonstrate robust convergence of estimated delays to ground truth (Wright, Zhang, et al., 2026).

**Fig. 1.**
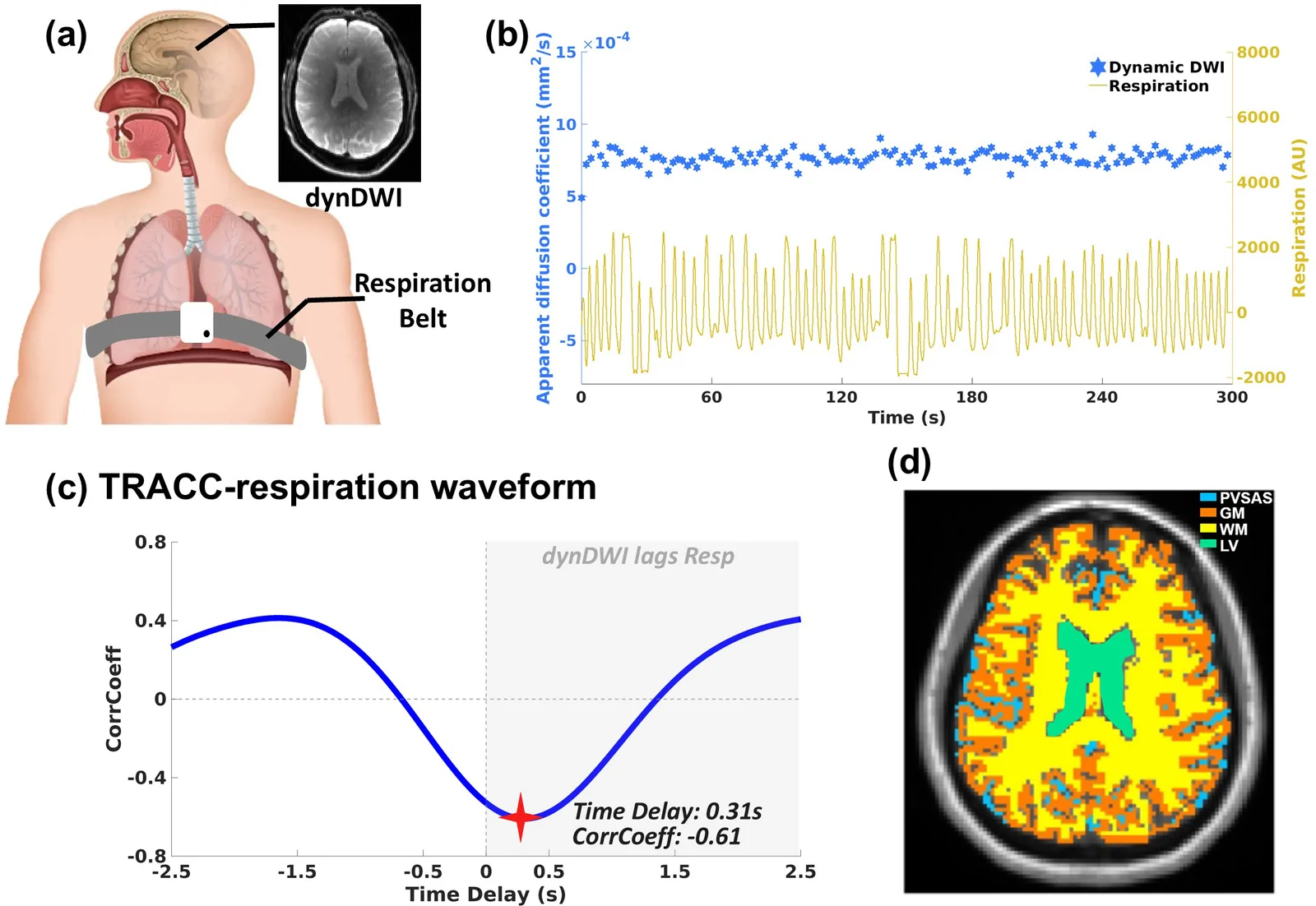
Overview of TRACC-respiration analysis. (a) Incoherent brain fluid dynamics was assessed using ADC from dynDWI, with simultaneous respiration recordings via a belt. (b) Time-domain signals for dynDWI across three diffusion directions (x, y, z), acquired in randomized order at 0.57 Hz (TR = 1750 ms) (example shown for GM), and respiration signals were sampled at 400 Hz. (c) TRACC-respiration waveform was computed between dynDWI and respiration signals across a time shift window of -2.5 to 2.5 seconds. The maximum CorrCoeff of -0.61 occurred at a 0.31-second TimeDelay, indicating a strong coupling between ADC variations and respiration, with brain fluid changes lagging respiration by 0.31 seconds. (d) Key regions of interest included the PVSAS, GM, WM, and LV. ADC, apparent diffusion coefficient; dynDWI, dynamic diffusion-weighted imaging; PVSAS, perivascular subarachnoid space; GM, gray matter; WM, white matter; LV, lateral ventricle; TRACC, Time-domain Resolution-Aligned Cross-Correlation.

In this framework, the dynDWI signal is iteratively shifted in 2.5-ms steps relative to the physiological waveform, and correlation is computed at each shift. This procedure was applied separately for each diffusion direction (x, y, z), and the per-direction TRACC waveforms were averaged to yield a mean TRACC waveform (e.g., TRACC-respiration or TRACC-cardiac) (Fig. 1a–c). Two metrics are derived from the TRACC waveform: (1) coupling coefficient (CorrCoeff), ranging from –1 to 1, indicating the strength of the coupling. Coupling strength was classified following (Cohen, 2013): |r| < 0.1, negligible; 0.1 ≤ |r| < 0.3, weak; 0.3 ≤ |r| < 0.5, moderate; and |r| ≥ 0.5, strong; and (2) TimeDelay, reflecting the relative arrival time of physiological impulses in the brain compared with the peripheral physiological recordings. To define a threshold for meaningful coupling, null distributions of CorrCoeff were generated by cross-correlating dynDWI with physiological recordings from mismatched participants.

We performed both ROI-wise and voxel-wise TRACC-PHYSIO analyses. ROI-based analysis facilitates visual inspections of waveform characteristics, while voxel-wise analysis provides spatial maps of coupling. For ROI-wise analysis, mean dynDWI signals were extracted from each axial slice within four key neurofluid compartments: gray matter (GM), white matter (WM), lateral ventricles (LV), and perivascular subarachnoid space (PVSAS) (Fig. 1d). GM, WM, and LV were segmented from T1-weighted images using Freesurfer’s recon-all, and registered to dynDWI space using FSL’s epi_reg. PVSAS was identified using a data-driven algorithm that detects subarachnoid CSF exhibiting cardiac pulsation (Wen et al., 2022). To mitigate partial volume effects between parenchymal tissue and adjacent subarachnoid CSF, we applied ADC-based thresholding: GM and WM masks excluded voxels with ADC values greater than 1000 mm²/s, while the PVSAS mask excluded voxels with ADC values lower than 1500 mm²/s. For ROI-wise analysis, TRACC waveforms were computed for each slice due to slice timing differences. These slice-wise results were then averaged within each subject to generate subject-level waveform for group-level analyses. Voxel-wise TRACC analyses yielded CorrCoeff maps for each participant, which were spatially normalized to MNI space using ANTs nonlinear registration and averaged to generate group-level coupling maps.

### 2.5 Auxiliary analysis

To assess whether observed dynDWI–respiration coupling was influenced by respiration-related head motion, we performed the TRACC-PHYSIO analysis in the extracerebral tissues (e.g., fat, skull). Minimal coupling with respiration was expected in this region, which would indicate that respiration-related motion had a negligible impact on the observed coupling results.

To evaluate whether respiration-related cerebral blood volume (CBV) changes could explain the observed respiration-coupled DWI dynamics, we performed numerical simulations using a two-compartment intravoxel incoherent motion (IVIM) framework (Le Bihan et al., 1988). In this model, the measured voxel signal was modeled as the sum of tissue and blood compartments, with a time-varying blood volume fraction f(t). Changes in f(t) can modulate the measured signal and introduce an apparent ADC fluctuation. Simulations were performed using physiologically plausible baseline blood volume fractions of 5% in GM and 2% in WM (Federau et al., 2012; Leenders et al., 1990), together with reported respiration-driven CBV fluctuations of Δf≈0.6%–1.1% (Söderström et al., 2025). The resulting CBV-induced ADC modulation was then compared with the experimentally observed ADC modulation across the respiratory cycle (see Supplementary Section 2 and Fig. S2 for detailed simulation setup).

Lastly, to assess whether dynDWI–respiration coupling depends on diffusion weighting, we acquired additional data from three subjects using higher b-values (b = 300 and 500 s/mm²), which more strongly suppress blood signals. We examined whether respiration- and cardiac-related coupling patterns observed at b150 were preserved at higher b-values, thereby testing whether b150 is sufficient to suppress blood contributions.

### 2.6 Statistical analysis

The normality of extracted parameters, CorrCoeff and TimeDelay, was assessed using the Shapiro–Wilk test. For normally distributed data, paired t-tests with Bonferroni correction were used to compare coupling strength across ROIs. For non-normally distributed parameters, the Wilcoxon signed-rank test was applied. A statistical significance threshold of *p* < 0.05 was applied to determine the presence of significant effects, providing a benchmark for interpreting the observed results.

## 3 Results

### 3.1 Strong and consistent respiratory coupling in the parenchyma

**Fig. 2.**
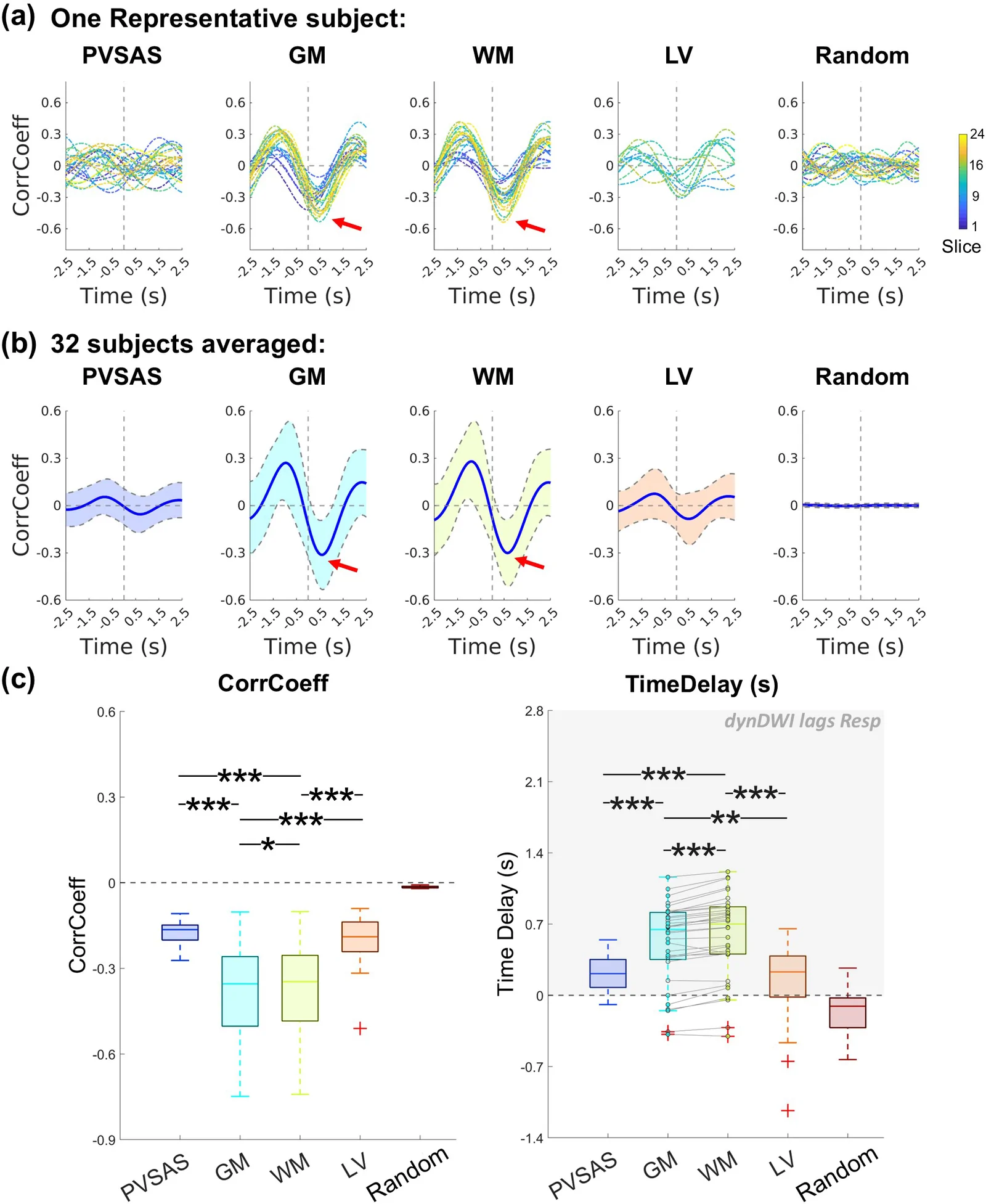
Strong and Consistent Respiratory Coupling in the Parenchyma. (a) TRACC-respiration waveforms from one representative subject showed consistent coupling across slices in GM (CorrCoeff = 0.41 ± 0.06). Each waveform represents a slice-wise TRACC waveform obtained by averaging across the three diffusion-encoding directions (x, y, z), for which TRACC waveforms were computed independently. The resulting slice-wise, direction-averaged waveforms allow visualization of coupling coherence across slices. (b) Group-averaged TRACC-respiration waveforms (N = 32) demonstrated a robust negative correlation between ADC fluctuations and the respiration signal. The shaded areas represent the standard error of the mean (SEM) across participants, where each participant contributed one waveform averaged across three diffusion-encoding directions and all slices. (c) Boxplots of CorrCoeff and TimeDelay across ROIs. The CorrCoeff was highest in GM, with the order of GM > WM > LV > PVSAS, all significantly higher than the random coupling (*p* < 0.001, paired t-test). The TimeDelay significantly differed among ROIs, with GM leading WM by 40 ms (*p* < 0.001, paired t-test). Paired GM and WM TimeDelay values for each subject are connected by lines to illustrate within-subject differences. *p < 0.05, **p < 0.01, and ***p < 0.001 in paired t-test with Bonferroni correction for multiple comparisons. TRACC, Time-domain Resolution-Aligned Cross-Correlation; CorrCoeff, cross-correlation coefficient; dynDWI, dynamic diffusion-weighted imaging; PVSAS, perivascular subarachnoid space; GM, gray matter; WM, whiter matter; LV, lateral ventricle; Random, randomized coupling generated by cross-correlating dynDWI signals with a mismatched respiration signal.

GM and WM exhibited stronger and more consistent TRACC-respiration coupling than PVSAS and LV. This trend was captured at both individual (one representative participant, Fig. 2a) and group levels (averaged across 32 subjects, Fig. 2b), as highlighted by red arrows. The coupling strength, quantified by median CorrCoeff (Q1 ~ Q3), followed the order among ROIs: GM: 0.37 (0.28 ~ 0.53) >WM: 0.36 (0.26 ~ 0.49) >LV: 0.21 (0.14 ~ 0.25) >PVSAS: 0.17 (0.15 ~ 0.22). All CorrCoeff values were significantly higher than the random coupling (*p* < 0.001, paired t-test with Bonferroni correction) (Fig. 2c). A maximum CorrCoeff of 0.77 was observed in GM for a single subject, indicating a strong respiratory influence on parenchymal fluid dynamics. The negative correlation coefficient indicated an inverse relationship between ADC fluctuations and respiration, specifically, ADC increased following exhalation (i.e., a decrease in belt signal), with a measurable temporal lag. The corresponding median TimeDelay (Q1 ~ Q3) was GM: 0.63 seconds (0.38 ~ 0.80), WM: 0.67 seconds (0.42 ~ 0.84), LV: 0.23 seconds (0.02 ~ 0.43), PVSAS: 0.25 seconds (0.12 ~ 0.36), all significantly different than random coupling (*p* < 0.001, paired t-test with Bonferroni correction) (Fig. 2c). The TimeDelay suggests the respiration-induced fluid dynamics first affect the LV and PVSAS, and subsequently propagate to the parenchyma. Notably, GM led WM by 40 ms, a difference that was statistically significant (*p* < 0.001, paired t-test with Bonferroni correction).

### 3.2 Distinct spatial patterns of respiration- and cardiac-coupled hydrodynamics

**Fig. 3.**
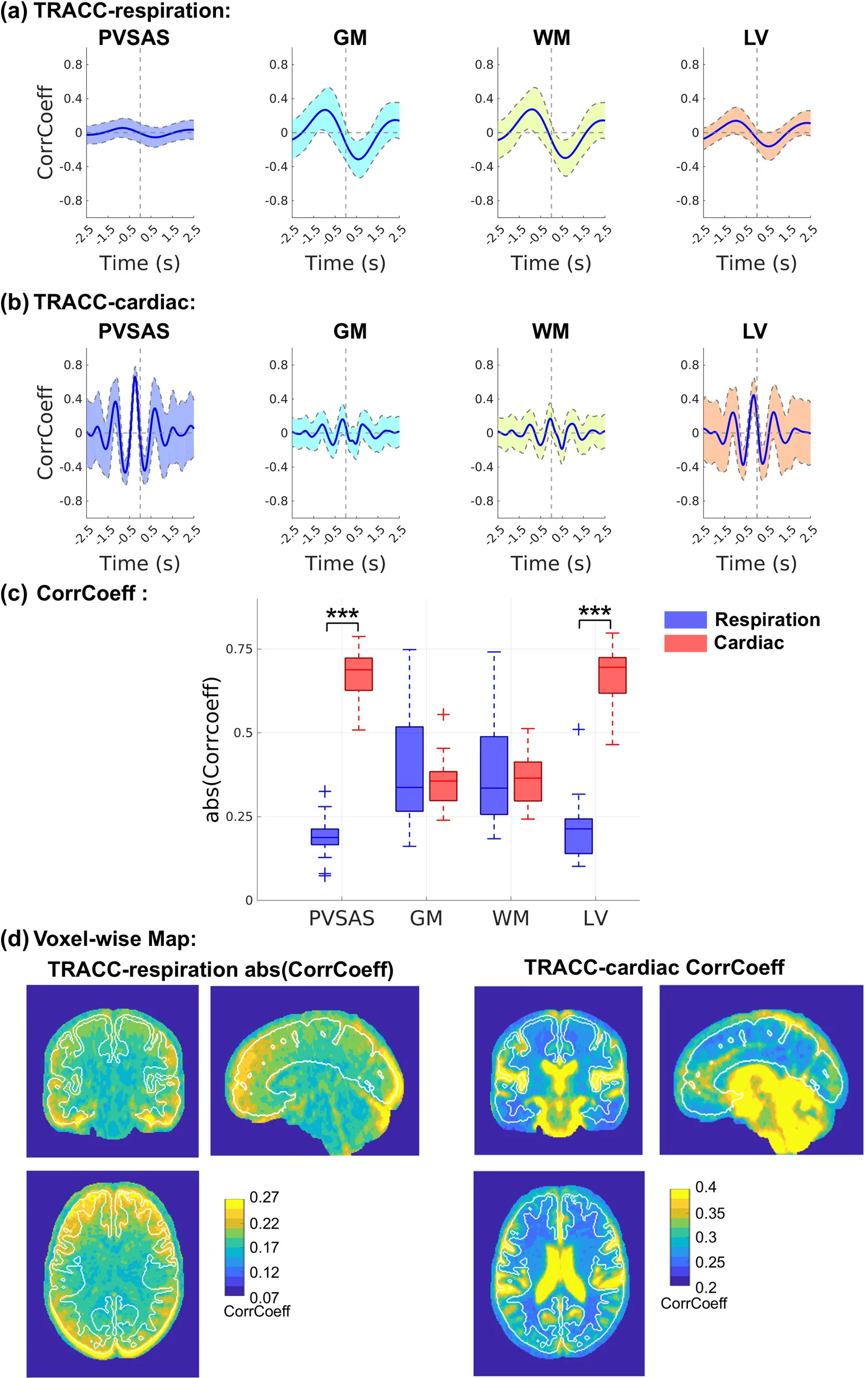
Distinct spatial patterns of respiration- and cardiac-coupled hydrodynamics. (a) TRACC-respiration coupling was higher in GM and WM. (b) TRACC-cardiac coupling was stronger in PVSAS and LV. The shaded areas in (a) and (b) represent the standard error of the mean (SEM) across participants, where each participant contributed one waveform averaged across all slices. (c) Boxplots of CorrCoeff illustrated that GM and WM had consistent couplings with both respiration and cardiac signals, while PVSAS and LV showed cardiac-dominant couplings. (d) Voxel-wise cross-correlation maps highlighted spatially distinct coupling patterns, with stronger respiration coupling in GM and stronger cardiac coupling in PVSAS and LV. Cortical contours are overlaid to delineate cortical regions. ***p < 0.001 in paired t-test with Bonferroni correction for multiple comparisons. TRACC, Time-domain Resolution-Aligned Cross-Correlation; CorrCoeff, cross-correlation coefficient; PVSAS, perivascular subarachnoid space; GM, gray matter; WM, whiter matter; LV, lateral ventricle.

The relative contributions of respiration and cardiac oscillations varied across brain regions. Respiration-coupled hydrodynamics were stronger in GM and WM than PVSAS and LV (Fig. 3a), as detailed in Section 3.1. In contrast, cardiac-coupled hydrodynamics were more prominent in PVSAS and LV, with median CorrCoeff (Q1 ~ Q3) reaching 0.72 (0.67 ~ 0.76) and 0.70 (0.62 ~ 0.73), respectively (Fig. 3b). Within the parenchyma, respiration and cardiac pulsation contributed similarly to its hydrodynamics (Fig. 3c). Voxel-wise CorrCoeff maps in MNI space further illustrated these distinct spatial patterns, with stronger respiration coupling in GM and stronger cardiac coupling in nonparenchymal regions, including PVSAS and LV (Fig. 3d).

More detailed voxel-wise (Supplementary Fig. S3) and lobe-wise analyses (Supplementary Table S2) revealed subtle regional differences, with stronger coupling and later arrival time in the frontal–temporal lobes than parietal–occipital lobes, suggesting a posterior-to-anterior propagation. No hemispheric differences were observed.

### 3.3 ADC fluctuations across respiration and cardiac cycles

**Fig. 4.**
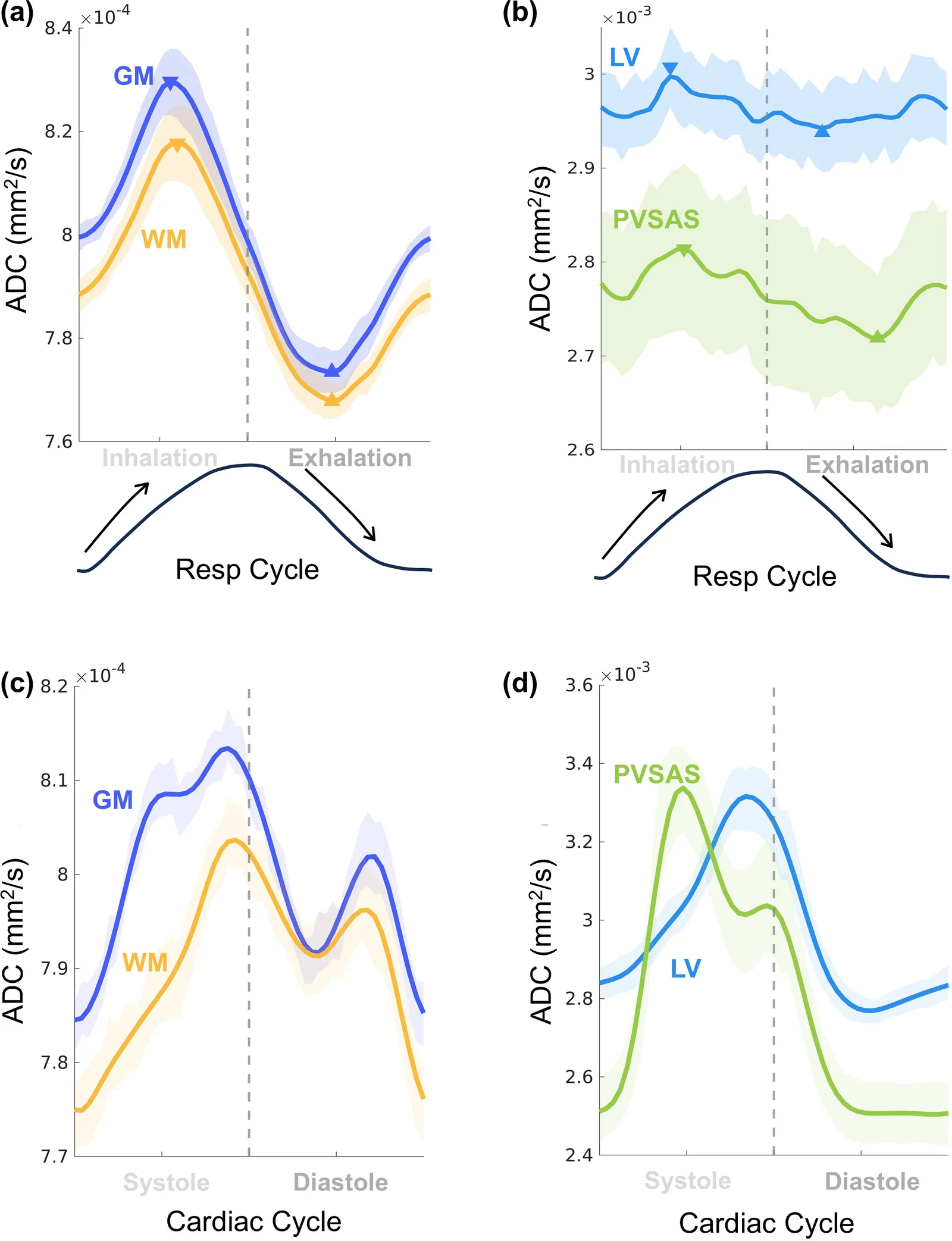
ADC fluctuations across the respiration and cardiac cycles. (a) ADC in GM peaked during inhalation (8.3 × 10^-4^ mm^2^/s) and reached a minimum during exhalation (7.7 × 10^-4^ mm^2^/s), corresponding to a 7% modulation. A similar pattern was observed in WM, with a temporal lag relative to GM. (b) Respiration-linked ADC modulations were 2% in LV and 4% in PVSAS. (c, d) During the cardiac cycle, ADC modulations were 4% in GM, 3% in WM, 28% in PVSAS, and 16% in LV. The shaded areas indicate mean ± SE across subjects. ADC, apparent diffusion coefficient; GM, gray matter; WM, white matter; PVSAS, perivascular subarachnoid space; LV, lateral ventricle.

Rebinning dynDWI time series into a single respiration cycle revealed that ADC in GM peaked during inhalation (8.3 × 10^-4^ mm^2^/s) and reached a minimum during exhalation (7.7 × 10^-4^ mm^2^/s), corresponding to a 7% modulation (Fig. 4a). ADC began to decrease shortly after inhalation and increased following exhalation, with a temporal delay (Fig. 4a). WM exhibited a similar pattern (8.1 × 10^-4^ to 7.7 × 10^-4^ mm^2^/s, reflecting a 6% modulation) (Fig. 4a), with a slight temporal lag relative to GM. These findings are consistent with the TRACC-respiration results (Fig. 2), indicating that parenchymal fluid responded to respiration with a delay and exhibited an inverse relationship. In contrast, respiration-linked ADC fluctuations were weaker in CSF regions, measuring 4% (2.8 × 10^-3^ to 2.7 × 10^-3^ mm^2^/s) in PVSAS and 2% (3.0 × 10^-3^ to 2.9 × 10^-3^ mm^2^/s) in LV (Fig. 4b).

Rebinning into a single cardiac cycle revealed cardiac-linked ADC modulations of 4% in GM (8.1 × 10^-4^ to 7.8 × 10^-4^ mm^2^/s) and 3% in WM (8.0 × 10^-4^ to 7.7 × 10^-4^ mm^2^/s) (Fig. 4c). In contrast, CSF compartments showed markedly larger cardiac modulations, with PVSAS showing 25% (3.3 × 10^-3^ to 2.5 × 10^-3^ mm^2^/s) and LV showing 16% (3.3 × 10^-3^ to 2.8 × 10^-3^ mm^2^/s) modulations (Fig. 4d).

### 3.4 The coupling is not dominant by hemodynamic effects and is consistent across b-values

The simulations showed that respiration-related CBV fluctuations produced only modest apparent ADC modulation. Within the physiologically plausible range of Δf≈0.6%–1.1% (Söderström et al., 2025), the simulated CBV-induced ADC modulation was only 0.35%–0.63% in GM and 0.17%–0.30% in WM (Supplementary Fig. S2). These values are nearly an order of magnitude smaller than the approximately 7% ADC modulation observed experimentally across the respiratory cycle. Thus, although CBV changes may contribute to the measured signal, blood volume fluctuations alone are insufficient to explain the observed ADC dynamics. These results support the interpretation that the respiration-coupled ADC fluctuations primarily reflect changes in parenchymal hydrodynamics rather than CBV dynamics alone.

**Fig. 5.**
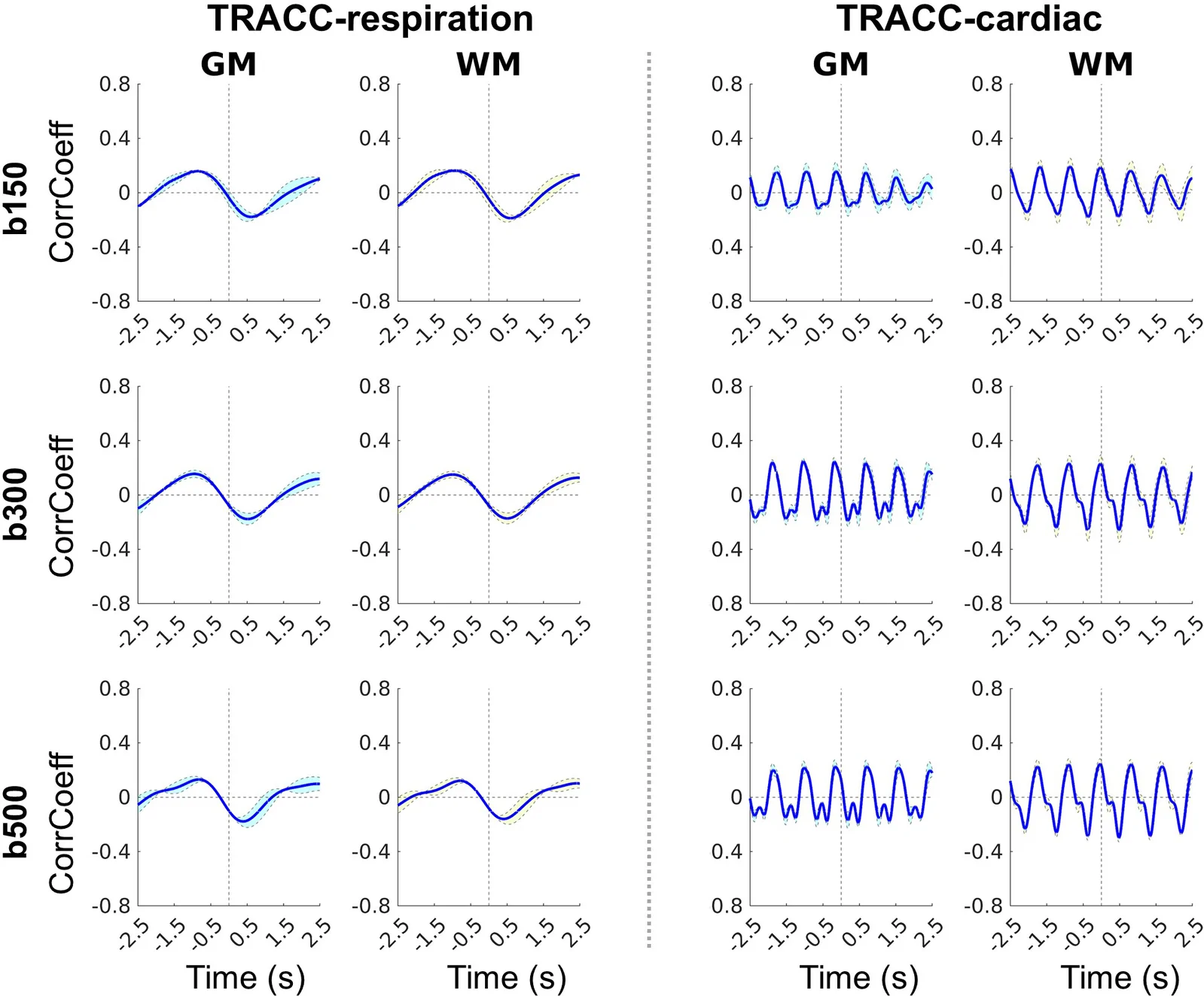
CorrCoeff computed from ADC time series, showing TRACC-respiration (left) and TRACC-cardiac (right) coupling across diffusion weightings (b = 150, 300, 500 s/mm²). Here, ADC is a derived quantity computed from the ratio of diffusion-weighted and b0 signals. The consistent coupling patterns across b-values indicate that the observed dynamics are preserved beyond low-b conditions. TRACC, Time-domain Resolution-Aligned Cross-Correlation; CorrCoeff, cross-correlation coefficient; GM, gray matter; WM, white matter.

Across all b-values (150, 300, and 500 s/mm²), respiration- and cardiac-coupling patterns were consistent (Fig. 5). This consistency across diffusion weightings suggests that b150 is sufficient to suppress blood-related contributions, comparable with higher b-values.

### 3.5 Additional results

#### 3.5.1 Respiration-related head motion

In the extracerebral tissues, TRACC-respiration coupling was low and inconsistent at both the subject and group levels (Supplementary Fig. S4), indicating that the observed parenchymal coupling is unlikely to be driven by respiration-related head motion.

#### 3.5.2 Impact of respiration and heart rate on coupling strength

Respiration rate was significantly associated with TRACC-Resp correlation coefficients, such that higher respiration rates corresponded to stronger ADC–respiration coupling in both GM and WM (p < 0.01 and p < 0.001, respectively; Bonferroni corrected) (Supplementary Fig. S5). In contrast, heart rate was not associated with TRACC-Cardiac coupling.

#### 3.5.3 Effect of diffusion-encoding direction

We further examined the influence of diffusion-encoding direction on coupling (Supplementary Fig. S6). Respiration coupling showed no differences in either CorrCoeff or TimeDelay across encoding directions (Supplementary Fig. S6a, b). In contrast, cardiac coupling exhibited direction dependence in both parenchyma and the lateral ventricle, with higher CorrCoeff along the y-direction in the lateral ventricle (Supplementary Fig. S6c). No significant differences in cardiac-related TimeDelay were observed across diffusion-encoding directions (Supplementary Fig. S6d).

## 4 Discussion

Using dynDWI, we assessed parenchymal hydrodynamics and their physiological drivers. Respiration-related coupling was stronger in GM and WM than in perivascular subarachnoid space and ventricles, highlighting respiratory as a driver of parenchymal fluid mobility. In contrast, cardiac coupling peaked in the subarachnoid space and lateral ventricles compared with GM and WM, revealing a spatial pattern distinct from respiration‐driven dynamics.

### 4.1 Specificity of dynDWI to hydrodynamics in brain parenchyma

This study represents a first application of dynDWI with low b-value (150 s/mm²) to investigate drivers of parenchymal fluid dynamics. The choice of b-value is critical for probing parenchymal fluid compartments, which are broadly categorized into (1) restricted intracellular and axonal water, (2) relatively free-moving perivascular CSF and ISF, and (3) fast-flowing blood. While high b-values (>800 s/mm²) are typically used to assess restricted diffusion in intracellular and axonal compartments, lower b-values (<800 s/mm²) are more suitable for detecting less restricted components such as CSF, ISF, and blood.

Capillary blood flow is considerably faster than perivascular CSF flow (~1 mm/s vs ~20 μm/s in the mouse brain) (Bedussi et al., 2018; Mestre et al., 2018) and can be effectively suppressed using b-values between 20 and 200 s/mm². Consistent with this, prior IVIM studies have shown that blood signal contribution becomes negligible (<0.1%) at b-value = 200 s/mm² (Le Bihan, 2019). This principle is also employed in diffusion-weighted arterial spin labeling (dw-ASL), where b-values of 20–100 s/mm² are used to suppress vascular signals (Kim, 2006; Shao et al., 2019; Silva et al., 1997; Wang et al., 2007). In our study, we selected a b-value of 150 s/mm², which is sufficient to suppress most fast blood flow, thereby enhancing sensitivity to the slower-flowing components such as perivascular CSF and ISF.

The minimal hemodynamic contribution at b150 is further supported by the IVIM-based simulation and by the experiment results at b300 and b500. The simulation showed that physiologically plausible respiration-driven CBV fluctuations would produce only 0.35%–0.63% ADC modulation in GM and 0.17%–0.30% in WM, nearly an order of magnitude smaller than the 7% ADC modulation observed experimentally. At higher diffusion weightings (b = 300 and 500 s/mm²), where perfusion-related blood signals are more strongly suppressed, both respiration- and cardiac-related coupling patterns remained consistent with those observed at b150. Together, these findings indicate that the physiological coupling observed at b150 primarily reflects diffusion-related hydrodynamics rather than perfusion-related hemodynamic contributions.

To further rule out potential artifacts, we evaluated alternative explanations, including respiratory-related head motion, magnetic field fluctuations, and tissue deformation. Respiratory motion artifacts were evaluated by testing whether rigid head motion or extracerebral (e.g., skull) signals were temporally coupled to respiration. Neither exhibited significant coupling, indicating that the observed respiration-related effects are unlikely driven by head motion. We also considered respiration-induced B_0_ field fluctuations associated with thoracic movements (van Gelderen et al., 2007; Windischberger et al., 2002). Several factors suggest that this effect is minimal in our data: (1) B_0_-related effects are typically global, whereas our results show regional specificity; (2) B_0_ fluctuations primarily affect the phase in T2*-weighted images, rather than the magnitude (Jaffray et al., 2024; Peters et al., 2019); and the spin-echo T2-weighted sequence further provides inherent robustness to B_0_ inhomogeneities. Tissue deformation is another possible source of ADC variation. However, displacement encoding with stimulated echoes (DENSE) MRI measurements indicates that respiration-induced brain strain is approximately one-third of cardiac-induced brain strain (Sloots et al., 2020), whereas respiration-coupled ADC modulation in our data was about twofold larger than cardiac-coupled modulation (~7% vs. ~4%). This mismatch suggests that tissue deformation alone is unlikely to explain the respiration-related ADC fluctuations. Together, these findings support the interpretation that low-b-value dynDWI primarily reflects respiration-driven physiological hydrodynamics in brain parenchyma, rather than perfusion-, motion-, field-, or deformation-related effects.

### 4.2 Direct respiratory modulation of parenchymal fluid dynamics

By sensitizing to fluid dynamics and applying the TRACC-PHYSIO method, our results revealed respiration-coupled dynamics with sub-second latencies, highlighting rhythmic parenchymal hydrodynamics directly synchronized with the respiratory cycle. While previous blood oxygenation level-dependent (BOLD) fMRI studies have reported respiration-related signal fluctuations, these typically reflect slower cerebrovascular responses to CO_2_ changes—linked to respiratory volume variability—occurring at lower frequencies (~0.03 Hz) with latencies of several seconds (Birn et al., 2008; Chang & Glover, 2009). In contrast, our observed dynamics occur at the respiratory rhythm (0.2–0.4 Hz) with a short latency (~0.7 seconds), suggesting a direct mechanical or pressure-mediated coupling rather than a vascular reactivity effect.

Recent studies using fast fMRI techniques such as magnetic resonance encephalography (MREG) have also reported respiration-synchronized signal oscillations within the 0.2–0.4 Hz range, prominently in the cortex, with centripetal propagation toward the brain center (Kiviniemi et al., 2016; Raitamaa et al., 2021; Syväoja et al., 2025). This propagation pattern is broadly consistent with the GM-to-WM trend observed in our dynDWI data. However, the spatial distribution and phase characteristics of respiratory coupling differ substantially between the two modalities. MREG studies report weaker respiratory coupling in the cortex and stronger coupling in the brainstem and ventricles, opposite to the distribution observed in our data, and show phase inversion between GM and WM signals (Kiviniemi et al., 2016; Raitamaa et al., 2021). In contrast, dynDWI exhibits coherent phase and consistent signal direction across GM and WM. These differences likely arise from distinct contrast mechanisms: MREG is T2*-weighted and primarily sensitive to BOLD effects, whereas dynDWI offers greater specificity to incoherent, nonvascular fluid motion within the parenchyma.

### 4.3 Distinct spatial coupling patterns of respiration and cardiac pulsation

Our results revealed a clear spatial dissociation between respiration- and cardiac-driven fluid dynamics. This spatial heterogeneity suggests that the driving forces differ by brain regions. The robust cardiac-driven CSF pulsation in the PVSAS is consistent with prior studies (Bedussi et al., 2018; Mestre et al., 2018; Wen et al., 2022, 2023, 2025). However, this pulsatile force appears to be substantially attenuated as it propagates into the brain parenchyma via penetrating arterioles, whose high compliance and downstream vascular resistance dampen high-frequency cardiac-driven pressure waves, therefore, protecting the delicate small vessels from mechanical stress and stabilizing cerebral blood flow (Nichols et al., 2022; Rey & Sarntinoranont, 2018).

In contrast, respiratory waves—approximately four times slower than cardiac pulsations—were effectively transmitted into the brain parenchyma, as evidenced by strong coupling in GM and WM. This suggests that respiration provides a complementary driving force to cardiac pulsation, contributing to parenchymal fluid mixing and potentially facilitating solute transport. These distinct spatial coupling patterns further reflect mechanical filtering: high-frequency waves such as cardiac pulsation are more strongly dampened by structural barriers, whereas lower-frequency respiratory forces propagate more readily and penetrate brain regions. This behavior is analogous to sound transmission, in which low-pitched sounds travel farther through barriers than high-pitched sounds.

Beyond these distinct spatial patterns, respiration- and cardiac-driven ADC changes also differed in their directional dependence. Cardiac-driven ADC fluctuations exhibited clear direction dependence, consistent with prior studies (Sloots et al., 2022; Surer et al., 2018; Wright, Wu, Feng, et al., 2024), whereas respiration-driven ADC changes showed little to no directional specificity. These observations suggest that respiration may exert a more spatially diffuse, low-frequency pressure effect that promotes fluid motion in a less directionally constrained manner, as discussed below.

### 4.4 Transmission pathways of respiratory forces to the parenchyma

One plausible transmission pathway of respiratory forces reaching the parenchyma involves hemodynamic mediation. PC MRI studies have demonstrated that during free breathing, net cerebral blood flow decreases during inhalation due to a combination of increased venous outflow and reduced arterial inflow (Liu et al., 2024, 2025; Söderström et al., 2025). This drop in cerebral blood volume creates spaces for the perivascular CSF and interstitial fluid, promoting their movement and mixing—a process that aligns with our observation of peaked ADC during inhalation. Within this hemodynamic pathway, the venous system may play a particularly important role. Inhalation generates negative intrathoracic pressure, increasing venous return and producing respiration-coupled venous pressure oscillations that propagate across the meninges and create transmeningeal pressure gradients. These gradients can drive intracranial CSF motion, including the respiration-synchronized aqueductal CSF dynamics observed with phase-contrast MRI (Dreha-Kulaczewski et al., 2015; Yildiz et al., 2017), and are consistent with the respiration-coupled parenchymal dynamics observed in this study.

An alternative, nonvascular pathway involves direct mechanical transmission of thoracic pressure through the spinal canal. Respiratory-driven pressure gradients can propagate cranially from the thoracic cavity via the spinal canal and foramen magnum, transiently altering intracranial pressure (ICP) (Hamilton et al., 1936; Zhang et al., 2004). These cyclic pressure changes during inhalation and exhalation may compress or decompress parenchymal tissue, thereby influencing the mobility of CSF and ISF within the brain.

In both scenarios, the observed respiration–parenchyma coupling likely reflects biomechanical force transmission, supported by the short latency (~0.7 seconds) and a GM-to-WM propagation pattern. The key distinction between these mechanisms is whether the force is transmitted indirectly via cerebral blood volume changes, or directly through cranially propagating pressure waves that mechanically stress the parenchyma. Future studies integrating dynDWI with blood flow measurements and examining their temporal coupling may help differentiate these pathways and clarify how respiration influences parenchymal fluid dynamics.

### 4.5 Limitations

One limitation in this work is the inter-individual differences in breathing patterns. Although we standardized physiological conditions, such as requiring all participants to remain awake with eyes open, differences in breathing rhythms and styles likely remained. Chest and abdominal breathing may have distinct coupling effects, but our use of a single belt on the lower chest prevented us from distinguishing between them. Future investigations should systematically assess how breathing patterns, including depth and style, influence respiratory coupling in order to better understand the association between respiration rate and coupling strength observed in this study.

Another limitation is that residual hemodynamic contributions, including time-varying CBV effects, cannot be fully excluded. Although our IVIM-based simulations indicate that physiologically plausible CBV fluctuations are too small to explain the observed ADC modulation, such contributions may be further minimized with optimized imaging approaches. For example, ADC dynamics could be estimated using phase-matched higher-b-value pairs, such as b = 300 and 500 s/mm², which may reduce sensitivity to CBV-related effects. However, this approach would require careful cardiac triggering and respiratory gating to match physiological phases across diffusion-weighted acquisitions. Future studies using gated multi-b-value acquisitions may further separate hydrodynamic and hemodynamic contributions.

While TRACC-PHYSIO provides a time-domain approach to quantify respiratory and cardiac contributions to fluid dynamics, its performance depends on the quality of the PPG signal. Approximately 7% of the data were excluded due to poor belt or PPG signal quality. Frequency-domain methods remain the ideal approach for quantifying physiological pulsations, and future technical advancements enabling imaging TRs below 0.3 seconds may allow direct confirmation of these findings using frequency-domain analysis.

## 5 Conclusion

Using dynDWI, we observed respiration-dependent modulation of parenchymal diffusivity, with inhalation reducing and exhalation enhancing parenchymal fluid dynamics. We further identified spatially distinct contributions of respiration and cardiac pulsation: respiratory modulation was stronger in the parenchyma than in the PVSAS and lateral ventricles, whereas cardiac pulsation predominated in the PVSAS and ventricles. Together, these patterns indicate that respiration and cardiac pulsation play complementary, region-specific roles in regulating brain fluid dynamics.

## Supplementary Material

Supplementary Material

## Data Availability

The original imaging data cannot be made available for public access due to ethical and privacy considerations. A demo code is available with the corresponding author through reasonable requests.
