# Supplementary material for: Dynamic DWI detects respiratory modulation of brain parenchymal hydrodynamics
Source: Imaging Neurosci (Camb). 2026 Sep 24;4:IMAG.a.1369. doi: 10.1162/IMAG.a.1369 (PMC13613705; doi:10.1162/IMAG.a.1369)
Supplement: Supplementary file 1 [file IMAG.a.1369_supp.pdf]

Supplementary Materials

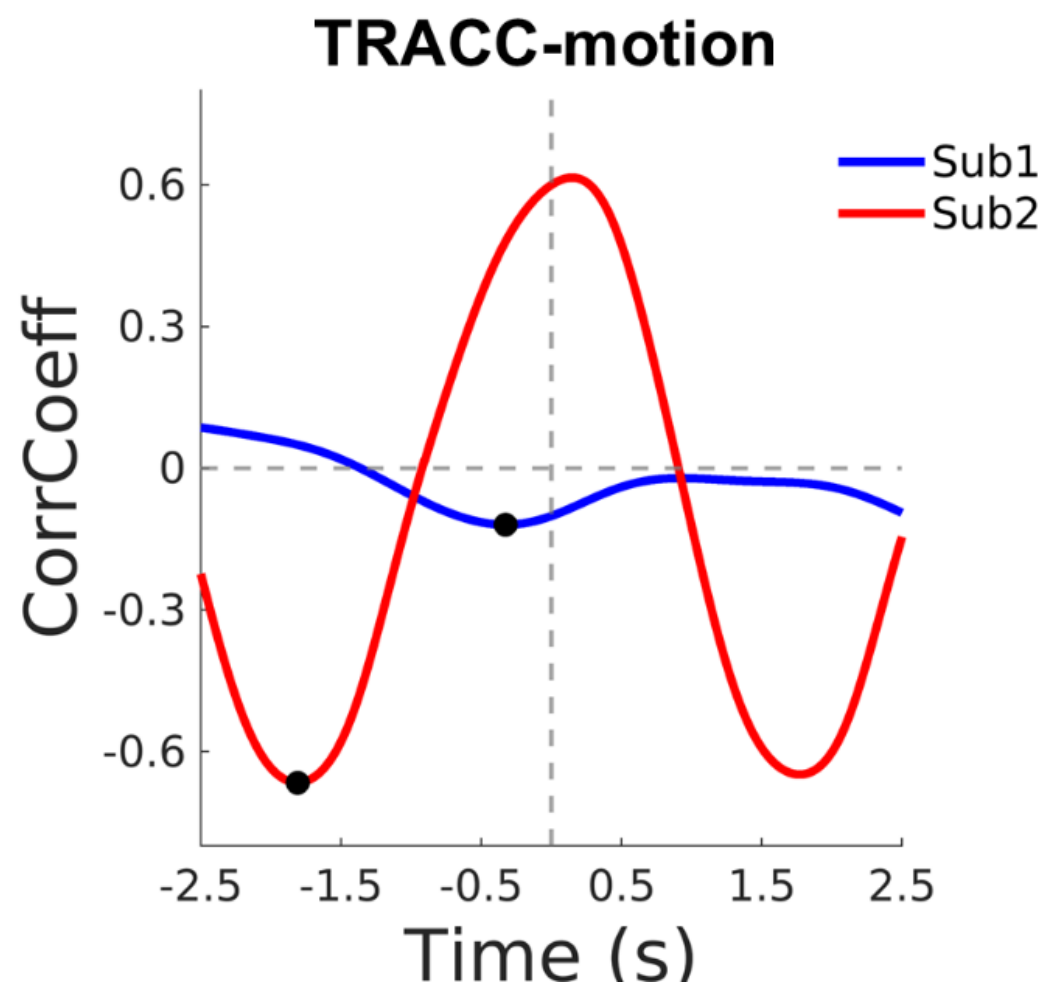

Figure S1. TRACC-motion waveforms in two representative subjects indicating low and high coupling between dynDWI motion (absolute displacement) and respiration. (a) Subject 1 showed low motion-respiration coupling, indicating minimal respiratory motion artifact. **(b)** Subject 2 demonstrated high motion–respiration coupling (|CorrCoeff| > 0.6), indicative of stronger respiratory-related head motion; this subject was excluded from the analysis. Abbreviations: TRACC, Time-domain Resolution-Aligned Cross-Correlation; Sub, subject; CorrCoeff, cross-correlation coefficient.

S2: Numerical simulation of CBV-induced ADC modulation using an IVIM model.

To evaluate whether respiration-related cerebral blood volume (CBV) changes could explain the observed ADC fluctuations, we performed numerical simulations using a two-compartment intravoxel incoherent motion (IVIM) model. In this framework, the measured signal in a voxel containing both tissue and blood can be expressed as:

$$S(b,t) = \big(1 - f(t)\big)S_{0_{tissue}}e^{-bD} + f(t)S_{0_{blood}}e^{-bD^*} \quad (1)$$

Where $f(t)$ is the time-varying blood volume fraction, $S_{0_{tissue}}$ and $S_{0_{blood}}$ are the baseline signals from pure tissue and pure blood, respectively. D is the non-blood fluid diffusion coefficient, and D^* is the pseudo-diffusion coefficient associated with incoherent blood motion.

For diffusion weighting of b≥150 s/mm2, the blood-related pseudo-diffusion component is expected to be largely suppressed. The signal can therefore be approximated as:

$$S(b \gg D^*, t) = \big(1 - f(t)\big)S_{0_{tissue}}e^{-bD} \quad (2)$$

b=0 signal is:

$$S(0,t) = \big(1 - f(t)\big)S_{0_{tissue}} + f(t)S_{0_{blood}} \quad (3)$$

Thus, even if the underlying non-blood fluid diffusion coefficient D remains constant, changes in $f(t)$ can introduce an apparent ADC fluctuation when ADC is estimated from the ratio of diffusion-weighted and b=0 signals:

$$ADC(t) = -\frac{1}{b}\left(\ln\left(\frac{(1-f(t))S_{0_{tissue}}}{(1-f(t))S_{0_{tissue}} + f(t)S_{0_{blood}}}\right) - bD\right) \quad (4)$$

Which simplifies to

$$ADC(t) \approx D - \frac{1}{b}\ln\left(\frac{(1-f(t))S_{0_{blood}}}{(1-f(t))S_{0_{tissue}} + f(t)S_{0_{blood}}}\right) \quad (5)$$

We used this formulation to simulate the expected ADC modulation caused solely by respiration-cycle–dependent changes in blood volume fraction, Δf. The baseline signal estimates were set to $S_{0_{blood}} = 0.40$, $S_{0_{tissue}} = 0.36$, based on representative 3T relaxation properties under our imaging parameters. Baseline blood volume fractions were set to 5% in gray matter (GM) and 2% in white matter (WM), based on literature estimates (Federau et al., 2012; Leenders et al., 1990).

We then simulated ΔADC as a function of Δf (Figure S3). Reported respiration-induced CBV variations Δf are approximately 0.6%-1.1% per cycle (Söderström et al., 2025). Within this physiological range, the CBV-induced ΔADC modulation was approximately 0.35%-0.63% in GM and 0.17%-0.30% in WM (Figure S3). These values are nearly an order of magnitude smaller than the 7% modulation observed experimentally in both GM and WM (manuscript Figure 4a).

These simulations indicate that respiration-related CBV fluctuations can contribute to apparent ADC modulation, but their expected magnitude is too small to explain the observed ADC dynamics. Under physiologically plausible assumptions, CBV changes alone account for only a small fraction (~8%) of the measured ADC modulation. The remaining signal variation is therefore more consistent with changes in the apparent non-blood fluid diffusion term, D(t), which we interpret as reflecting respiration-associated hydrodynamic motion of CSF/interstitial/parenchymal fluid rather than CBV dynamics alone.

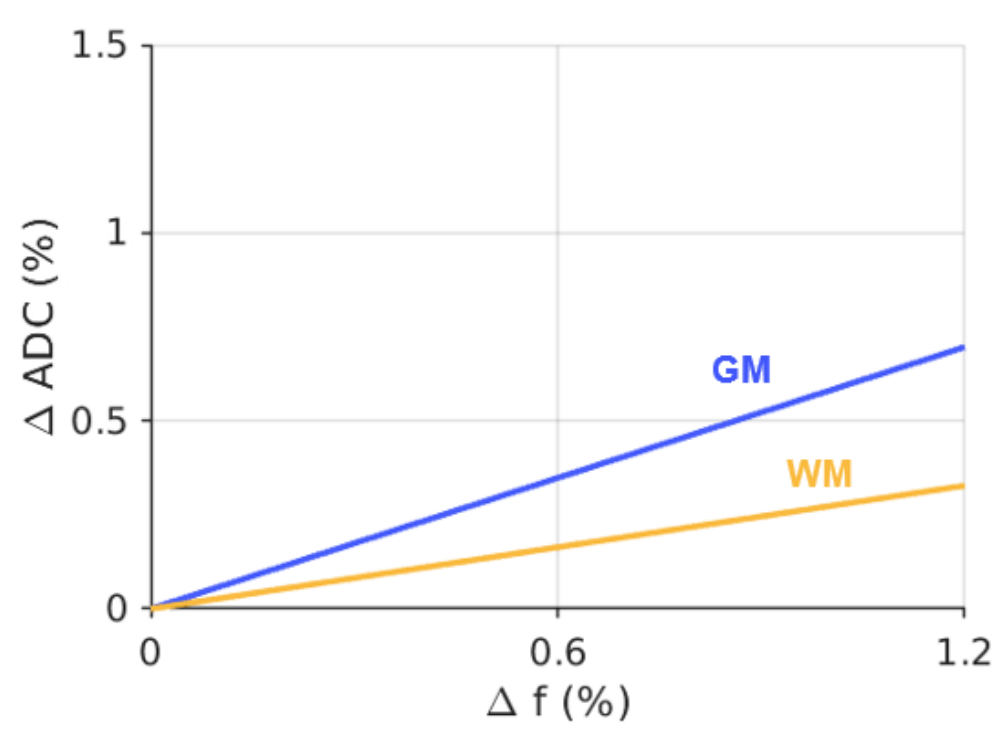

Figure S2. Simulation analysis of CBV-induced ADC modulation based on the two-compartment IVIM model. Relative changes in ADC (ΔADC) are plotted as a function of respiration-driven blood volume fraction fluctuations (Δf), calculated using equation (5). Within the physiologically plausible respiration-driven fluctuations in CBV (Δf ≈ 0.6%–1.1%), the resulting ADC modulation is approximately 0.35–0.63% in GM and 0.17–0.30% in WM—nearly an order of magnitude smaller than the 7% variation observed experimentally. Thus, CBV fluctuations alone can explain only a small fraction of the measured ADC dynamics, supporting the conclusion that the observed modulation is not dominated by CBV effects.

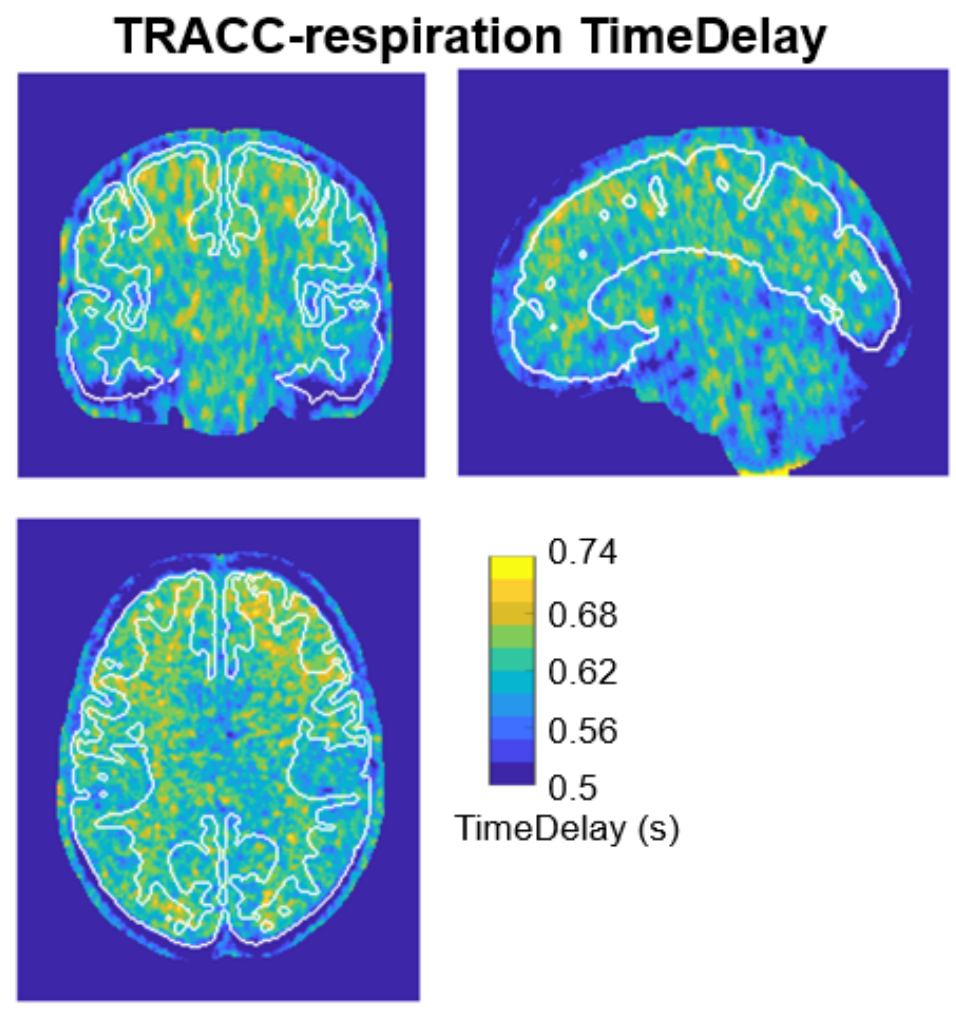

Figure S3. Voxel-wise TimeDelay Maps of TRACC-respiration. Abbreviations: TRACC, Time-domain Resolution-Aligned Cross-Correlation.

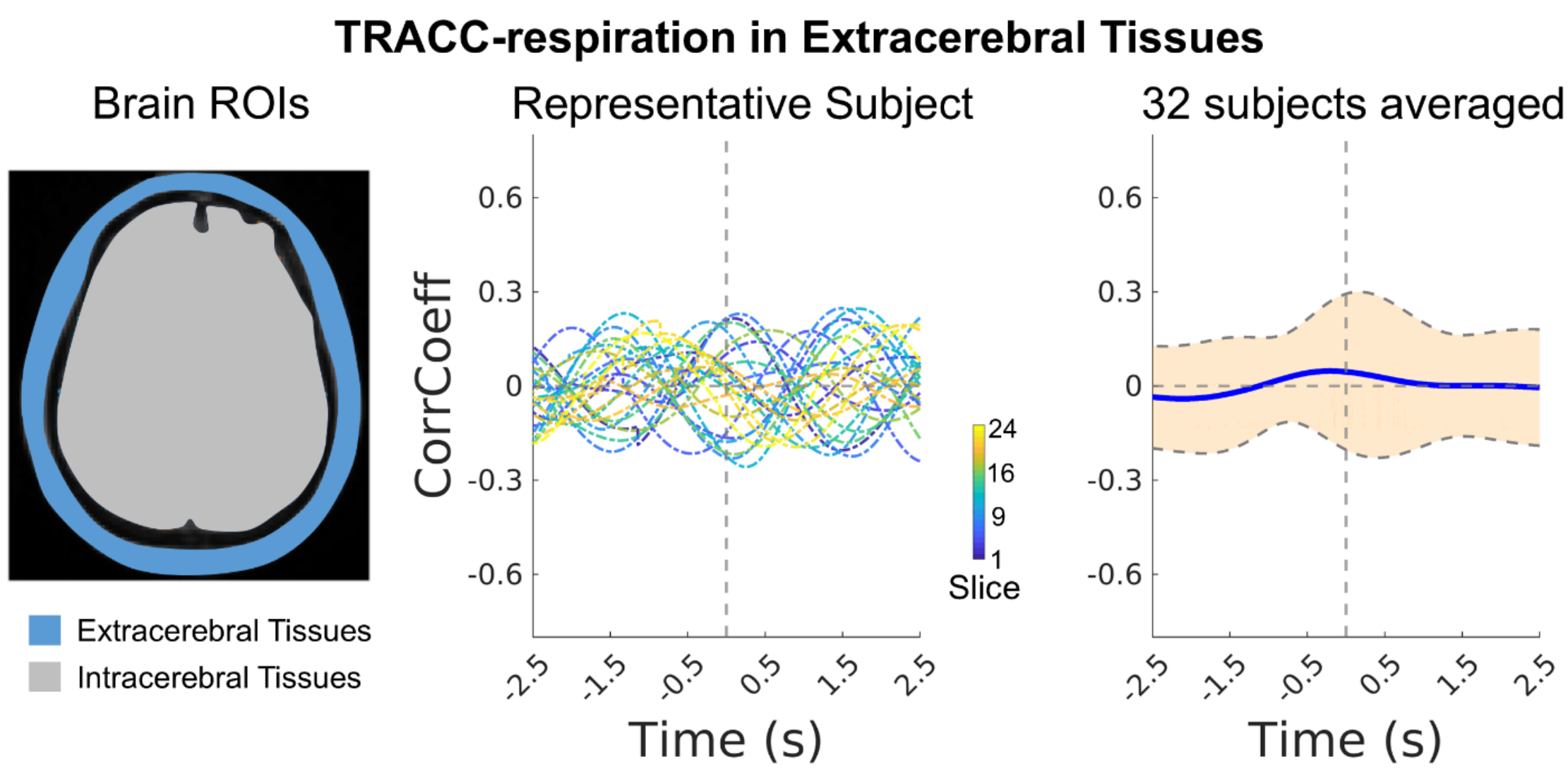

Figure S4. Minimal influence from respiratory-related motion on dynDWI signals. (a) a representative subject showed low and random CorrCoeff values for TRACC-respiration in extracerebral tissues (e.g., fat, skull). **(b)** group-mean CorrCoeff values for respiratory remained low, indicating minimal coupling. Abbreviations: TRACC, Time-domain Resolution-Aligned Cross-Correlation; Resp, respiration; CorrCoeff, cross-correlation coefficient.

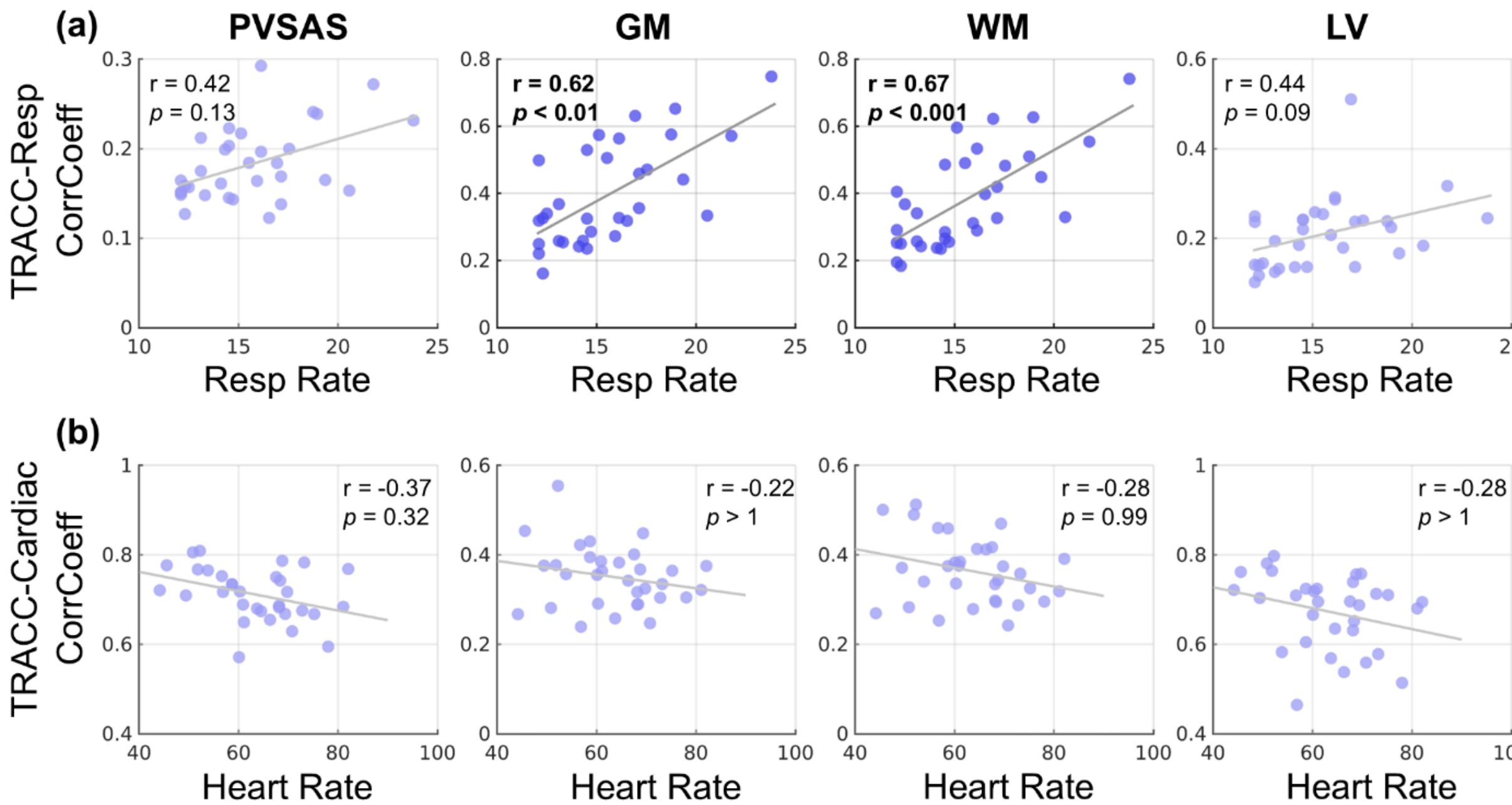

Figure S5. Correlations between physiological rates and coupling strength. (a) TRACC–Resp CorrCoeff increases with respiration rate in GM and WM, indicating that respiration rate contributes to the strength of respiration–ADC coupling. **(b)** No significant relationships were observed between heart rate and TRACC–Cardiac CorrCoeff across the four brain ROIs. Statistical tests were performed with Bonferroni correction. Abbreviations: TRACC, Time-domain Resolution-Aligned Cross-Correlation; Resp, respiration; CorrCoeff, cross-correlation coefficient; GM, gray matter; WM, white matter; LV, lateral ventricle; PVSAS, perivascular subarachnoid space.

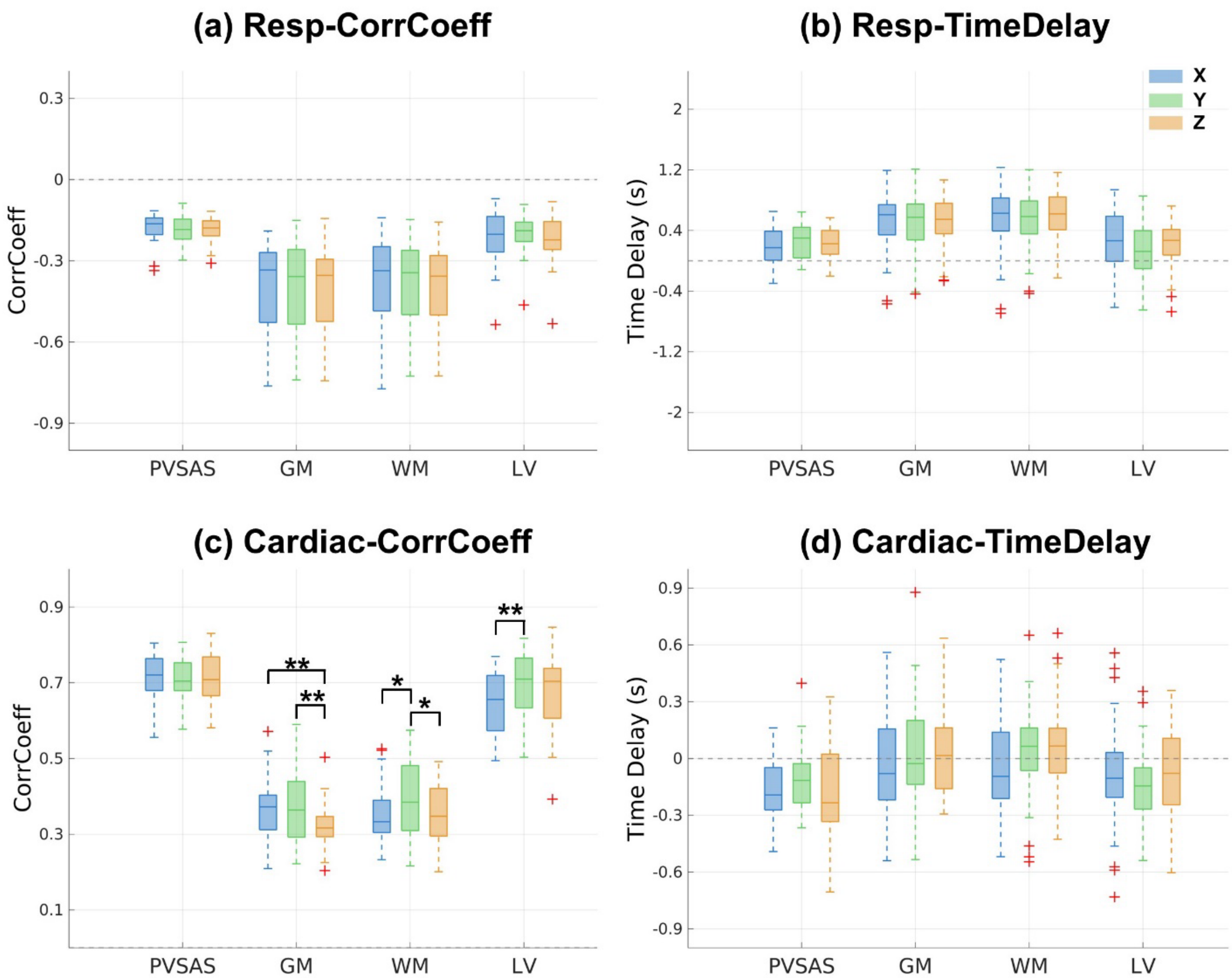

Figure S6. Direction-specific physiological coupling between ADC and respiration/cardiac signals. (a-b) CorrCoeff and TimeDelay of TRACC-respiration across three diffusion-encoding directions (x, y, z). No significant differences were observed across directions for either metric. **(c-d)** Cardiac-related CorrCoeff and TimeDelay across diffusion-encoding directions. The CorrCoeff showed significant direction dependence in parenchymal regions (GM and WM) and in LV, suggesting that cardiac coupling strength can vary with diffusion-encoding direction, whereas TimeDelay did not differ significantly. (*$p < 0.05$, **$p < 0.01$; paired t-test/rank test). Abbreviations: TRACC, Time-domain Resolution-Aligned Cross-Correlation; Resp, respiration; CorrCoeff, cross-correlation coefficient; GM, gray matter; WM, white matter; LV, lateral ventricle; PVSAS, perivascular subarachnoid space.

Table S1: The demographic information of the collected participants.

Subject	Age	Sex	Handedness	Heart rate (bpm)	Respiration rate (bpm)
S1	35	1	1	56.4	14.4
S2	35	1	2	81.0	14.4
S3	36	2	1	66.6	12.0
S4	36	1	1	61.2	12.6
S5	36	2	1	75.0	18.6
S6	36	2	1	58.8	12.0
S7	42	2	3	69.6	13.2
S8	42	1	1	82.2	12.0
S9	42	2	1	51.6	16.2
S10	43	1	1	45.6	14.4
S11	45	2	1	72.6	17.4
S12	46	2	1	64.8	16.2
S13	48	2	1	69.0	19.2
S14	53	1	1	63.6	17.4
S15	55	2	1	69.6	19.2
S16	55	1	1	51.0	24.0
S17	55	2	1	58.8	13.2
S18	56	2	2	78.0	15.0
S19	57	2	1	68.4	12.6
S20	59	2	1	60.6	12.0
S21	60	2	1	67.8	20.4
S22	60	1	1	60.0	16.2
S23	61	2	1	68.4	13.2
S24	62	2	1	70.8	21.6
S25	63	2	1	52.2	12.6
S26	63	1	2	54.0	17.4
S27	64	2	1	49.2	16.8
S28	64	2	1	61.2	15.0
S29	68	2	1	73.2	14.4
S30	72	2	1	68.4	14.4
S31	74	1	1	44.4	15.6
S32	82	2	2	57.0	16.8
Metric	Mean: 53.3<br>SD: 12.7	Male:10 (31%)<br>Female: 22 (69%)	R: 27 (84%), L: 4 (13%)<br>Ambidextrous: 1 (3%)	Mean: 63.5<br>SD: 9.7	Mean: 15.6<br>SD: 3.0

Note: Sex: 1 = male, 2 = female; Handedness: 1 = right, 2 = left, 3 = ambidextrous. SD: standard deviation; bpm: beat per minute.

Table S2: Regional differences in TRACC-respiration coupling across lobes, revealing stronger coupling and longer delays in frontal-temporal regions compared with posterior lobes.

Tissue	Lobe	CorrCoeff (Mean±SD)	TimeDelay (s) (Mean±SD)
GM	Frontal	-0.31±0.04	0.50±0.06
	Parietal	-0.28±0.02	0.43±0.06
	Temporal	-0.31±0.05	0.41±0.07
	Occipital	-0.25±0.04	0.31±0.04
WM	Frontal	-0.31±0.03	0.54±0.05
	Parietal	-0.28±0.01	0.46±0.05
	Temporal	-0.28±0.02	0.44±0.05
	Occipital	-0.25±0.01	0.36±0.04

Note: GM: gray matter; WM: white matter; CorrCoeff, cross-correlation coefficient; SD: standard deviation.

Reference

Federau, C., Maeder, P., O'Brien, K., Browaeys, P., Meuli, R., & Hagmann, P. (2012). Quantitative measurement of brain perfusion with intravoxel incoherent motion MR imaging. *Radiology*, *265*(3), 874-881.

Leenders, K. L., Perani, D., Lammertsma, A. A., Heather, J. D., Buckingham, P., Healy, M. J., Gibbs, J. M., Wise, R. J., Hatazawa, J., Herold, S., & et al. (1990). Cerebral blood flow, blood volume and oxygen utilization. Normal values and effect of age. *Brain*, *113 (Pt 1)*, 27-47. https://doi.org/10.1093/brain/113.1.27

Söderström, P., Eklund, A., Karalija, N., Andersson, B. M., Riklund, K., Bäckman, L., Malm, J., & Wåhlin, A. (2025). Respiratory influence on cerebral blood flow and blood volume–A 4D flow MRI study. *Journal of Cerebral Blood Flow & Metabolism*, *45*(8), 1531-1542.
